# Continuous glucose monitoring profiles during postpartum glucose tolerance test results among people with gestational diabetes

**DOI:** 10.1002/pmf2.70143

**Published:** 2025-10-27

**Authors:** Alyssa R. Hersh, Alexandra C. Gallagher, Lucy Ward, Christian Huertas‐Pagán, Monica Rincon, Amy M. Valent

**Affiliations:** ^1^ Division of Maternal‐Fetal Medicine Department of Obstetrics & Gynecology Oregon Health & Science University Portland Oregon USA; ^2^ Division of Maternal‐Fetal Medicine Department of Obstetrics & Gynecology Stanford University Stanford California USA; ^3^ OB/GYN Biostatistics Core Department of Obstetrics & Gynecology Oregon Health & Science University Portland Oregon USA

**Keywords:** continuous glucose monitoring, gestational diabetes, glucose tolerance test, postpartum, pregnancy

## Abstract

**Objective:**

Current guidance recommends universal screening with a postpartum oral glucose tolerance testing (OGTT) 4–12 weeks following delivery among pregnancies complicated by gestational diabetes mellitus (GDM) to identify or assess risk for prediabetes and type 2 diabetes (T2D). Continuous glucose monitoring (CGM) is not currently a tool for screening for T2D among pregnant or postpartum people. Our objective was to assess CGM data during the postpartum OGTT.

**Methods:**

This was a secondary analysis of a trial that randomized people with GDM to CGM versus capillary blood glucose (CBG). Participants were included if CGM data were available during their OGTT. Our primary outcome was mean glucose during the postpartum OGTT (75 g 2‐h challenge), comparing those with dysglycemia on OGTT (fasting ≥100 mg/dL and/or 2‐h ≥140 mg/dL) to normal OGTT results.

**Results:**

A total of 51 patients met the inclusion criteria for this secondary analysis. Mean CGM glucoses were higher at all time points for those with dysglycemia compared with a normal OGTT result. Among those with dysglycemia, the mean fasting plasma glucose was 104.3 ± 10.5 and the mean 2‐h plasma glucose was 129.9 ± 37.5. For those with normal OGTT results, the mean fasting plasma glucose was 89.8 ± 5.2 and the mean 2‐h plasma glucose was 103.5 ± 17.9. When stratified by GDM type, mean CGM glucose was similar between groups.

**Conclusion:**

In this study, we found that glucose measured by CGM follows the expected curve in response to the postpartum OGTT among individuals with pregnancies complicated by GDM. Those with dysglycemia had higher mean CGM glucose at all time points assessed. Future studies will need to assess the role of CGM in risk stratifying those with dysglycemia postpartum without an OGTT.

## 1 BACKGROUND

Gestational diabetes mellitus (GDM) is a condition specific to pregnancy with long‐term health implications. Recent studies have estimated that the risk of developing type 2 diabetes (T2D) is 11–61 times higher among patients with GDM than those without GDM [1]. To identify patients with persistent metabolic dysfunction in the postpartum period, current guidelines recommend performing an oral glucose tolerance test (OGTT) 4–12 weeks after delivery [2]. While most people with GDM will have normal postpartum testing on the OGTT, high rates of elevated results consistent with prediabetes or overt T2D have been demonstrated, emphasizing the importance of early identification and treatment [3, 4]. Unfortunately, less than 50% of patients present for their postpartum OGTT, representing a critical missed opportunity to diagnose abnormal glycemia and intervene to prevent downstream complications [3, 5, 6].

Continuous glucose monitoring (CGM) is a tool that consists of a wearable device that provides frequent interstitial glucose readings, providing real‐time glycemic data. It is not currently used as a diagnostic tool for GDM or T2D [7]. Furthermore, there are scarce data regarding the use of CGM during the postpartum period for those with GDM, and there are no data on CGM glycemic profiles of GDM patients at the time of postpartum OGTT via CGM. Therefore, we compared CGM profiles during the postpartum OGTT (75 g, 2‐h challenge) among postpartum people who had normal to those with elevated OGTT results. We hypothesize that CGM can distinctly characterize hyperglycemia among people with abnormal glucose testing compared with those who have normoglycemia during the OGTT. Characterizing CGM profiles during an OGTT is an important initial step to understand the role of CGM to identify at‐risk individuals postpartum who would benefit from a more intensive, health optimization plan to reduce the progression to T2D.

## 2 METHODS

This was a secondary analysis of pregnant people with GDM randomized to using CGM (Dexcom G6) versus capillary blood glucose (CBG) for management of GDM [8]. In the parent trial, pregnant persons diagnosed with GDM using the 1‐step IADPSG or 2‐step Carpenter–Coustan criteria were eligible. Pregnant persons with multifetal gestation, adhesive allergy, use of chronic immunosuppressive medications, or pre‐existing diabetes were excluded. All participants in the parent trial were recommended to complete the 75 g 2‐h OGTT during the postpartum period and instructed to place a blinded Dexcom G6 Pro the morning before testing. For this secondary analysis, we included participants with CGM data during the postpartum OGTT. This study was conducted at Oregon Health & Science University (OHSU) and was approved by the Institutional Review Board at OHSU (IRB #21775).

Our primary outcome was mean CGM glucose during the 75 g 2‐h OGTT, comparing participants who had dysglycemia on OGTT compared with normal OGTT results. The thresholds used to determine dysglycemia included a fasting plasma glucose ≥100 mg/dL (impaired fasting tolerance) and/or ≥140 mg/dL (impaired glucose tolerance) at the 2‐h measurement after 75 g glucose load. Participants were categorized in the dysglycemic group, if they had elevations above the normal range for one or both the fasting and 2‐h blood draws, and normoglycemia, if both values were within normal range. We additionally performed a stratified analysis by type of GDM during the pregnancy (A1—medical nutrition therapy and lifestyle behavioral management vs. A2—pharmacotherapy).

The CGM used in this study provides a glucose reading every 5 min. In our figures, fasting glucose was calculated by averaging all CGM readings in the hour prior to the fasting blood draw, and subsequent values averaged CGM readings over half‐hour time periods. These values are displayed in the figures with comparisons between glucose tolerance testing (GTT) outcomes and GDM type. Study data were maintained in REDCap hosted at OHSU. All data analyses were performed using R statistical software (v4.2.1; R Core Team 2022). All data were collected prospectively or through chart abstraction. Continuous data were analyzed using *t*‐tests and categorical data were assessed using chi‐square or Fisher's exact tests. We performed a sensitivity analysis assessing those who were excluded from this subanalysis, given they did not complete the postpartum OGTT or were not wearing a CGM during the OGTT. The age of participants included in our study was significantly higher than those who were excluded, but other factors were not significantly different between those who were included and excluded (Table S1).

## 3 RESULTS

Of the 111 participants in the parent trial, we included 51 participants (*n* = 39 GDMA2 and *n* = 12 GDMA1) who had postpartum CGM data available at the time of OGTT for this secondary analysis; Figure S1. Among participants in this secondary analysis, the mean age was 34.7 years, and most participants were multiparous, had higher body mass index (BMI) ≥30, were lactating, and were privately insured Table 1. The postpartum OGTT was performed at a median of 42 (interquartile range [IQR] 34.5–49.5) days following delivery. Of the 51 participants, there were 20 (39%) participants who had dysglycemia and 31 (61%) participants who had a normal result on the 2‐h OGTT. None of the participants were using any antidiabetic medications at the time of OGTT.

**FIGURE 1:**
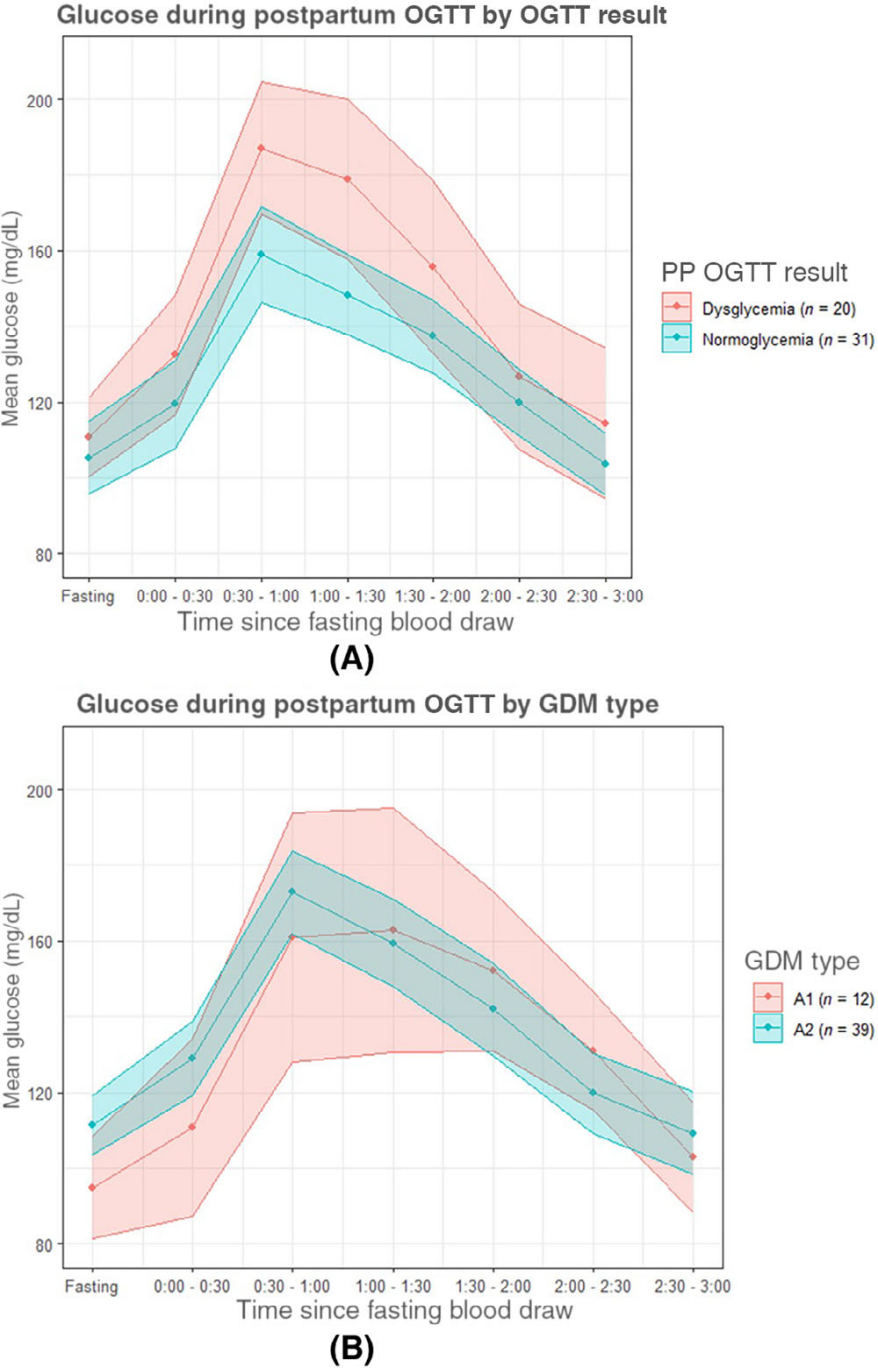
Mean glucose measurements with CGM during OGTT (start 1 h before fasting blood draw to 1 h after 2‐h blood draw; figure demonstrates mean value at 30‐min time increments with 95% confidence intervals). (A) Dysglycemia versus normoglycemia OGTT result; (B) A1GDM versus A2GDM. CGM, continuous glucose monitoring; GDM, gestational diabetes mellitus; OGTT, oral glucose tolerance testing.

For participants with dysglycemia on the postpartum OGTT, the mean fasting plasma glucose was 104.3 ± 10.5 mg/dL and the mean 2‐h plasma glucose was 129.9 ± 37.5 mg/dL. Among those with a normal postpartum OGTT result, the mean fasting plasma glucose was 89.8 ± 5.2 mg/dL and the mean 2‐h plasma glucose was 103.5 ± 17.9 mg/dL. At each half‐hour of measurement before, during, and after the postpartum OGTT, the mean interstitial glucose measured on CGM was higher for the group with dysglycemia compared with normoglycemia using OGTT (Figure 1A). When stratified by GDM type, mean CGM glucose was similar between groups (Figure 1B).

## 4 CONCLUSION

We found that mean CGM glucose profiles were higher among people with dysglycemia compared with those with a normal glucose response to the postpartum OGTT. The mean glucose calculated by CGM aligned with serum OGTT results, suggesting that CGM can distinctly characterize dysglycemia among those with an abnormal OGTT from those with a normal response. Participants with dysglycemia had a distinctly higher CGM glycemic pattern at all time points compared with those with a normal postpartum OGTT result.

Studies have shown low adherence to the recommendation for universal screening with the postpartum OGTT when completed after hospital discharge [5]. While studies have shown that a portion of screenings is missed due to provider‐related factors like failure to offer/order the OGTT, numerous other barriers and patient factors *also* influence compliance with postpartum OGTT. Time to perform an OGTT may require missed time from work, coordination for childcare assistance, and fasting overnight may be insurmountable for many patients who are sleep deprived, breastfeeding, and transitioning to parenthood or family expansion. CGM may be a tool to risk stratify those with prediabetes or T2D following delivery, as it provides useful glycemic data during a time when the hemoglobin A1C is not reliable. However, data regarding CGM glycemic patterns during the postpartum period and OGTT are scarce. Postpartum CGM profiling is needed to determine whether glycemic profiles can be distinguished without a glucose challenge. Specifically, future studies should investigate normative data in the postpartum period to determine an appropriate glucose range and derive postpartum metrics such as time‐in‐range and time‐above‐range that could be used to determine clinical risk and help patients have a long‐term goal for risk reduction.

**TABLE 1:** Participant characteristics overall, then stratified by normal versus abnormal GTT.

	Combined sample (*n* = 51)	Abnormal GTT (*n* = 20)	Normal GTT (*n* = 31)
GDM type			
A1	12 (23.5)	3 (15.0)	9 (29.0)
A2	39 (76.5)	17 (85.0)	22 (71.0)
Age	34.7 ± 3.9	35.9 ± 4.5	34.0 ± 3.35
Nulliparous (*n* [%])	19 (37.3)	5 (25.0)	14 (45.2)
BMI ≥ 30 kg/m^2^ (*n* [%])	31 (60.8)	16 (80.0)	15 (48.4)
Insurance (*n* [%])			
Private	35 (68.6)	13 (65.0)	22 (71.0)
Public/other	16 (31.4)	7 (35.0)	9 (29.0)
Early HbA1C	5.4 ± 0.5	5.6 ± 0.6	5.2 ± 0.3
Days from delivery to GTT	44.0 ± 17.4	43.5 ± 13.5	44.4 ± 19.7
Days from delivery to GTT (median [IQR])	42 [34.5‐49.5]	41.5 [37.5‐48.8]	42 [32.5‐50]
Breastfeeding at the time of GTT (*n* [%])	39 (76.5)	16 (80.0)	23 (74.2)
Pregnancy mean glucose	103.3 ± 14.0	105.3 ± 18.8	102.0 ± 9.7
% Pregnancy TIR (63–140 mg/dL)	91.6 ± 11.6	89.5 ± 15.9	93.1 ± 7.2
% Pregnancy TAR (>140 mg/dL)	7.1 ± 11.7	9.5 ± 16.0	5.4 ± 7.3

*Note*: Data presented in mean ± SD unless otherwise stated.

Abbreviations: BMI, body mass index; GDM, gestational diabetes mellitus; IQR, interquartile range; OGTT, oral glucose tolerance test; TAR, time above range; TIR, time in range.

This study contributes to the limited data available on postpartum CGM profiles. The parent trial was performed at a single center, which limits generalizability to broader populations. However, our rates of abnormal GTTs are similar to other reports, and we found a clinically relevant difference in glucose among those with dysglycemia compared with those with normal OGTT results [2]. Our available CGM data are limited to a short time period (day before to just after OGTT completion), and additional CGM output before and after the OGTT would have provided more data broadly regarding the utility of CGM for postpartum glycemic evaluation, which is a consideration for future studies. Lastly, given this was a secondary analysis, the study was not powered to look at differences in postpartum glycemic profiles between participants, and less than half of the study participants were eligible for inclusion, given non‐completion of the OGTT, which could bias the results. Given the importance of identifying alternative methods of assessing postpartum dysglycemia, future studies understanding the role of CGM use postpartum for prediabetes and T2D risk stratification are needed.

## CONFLICT OF INTEREST STATEMENT

The authors declare no conflicts of interest.

## FUNDING INFORMATION

The authors received no specific funding for this work.

## ETHICS STATEMENT

This study was conducted at Oregon Health & Science University (OHSU). The parent trial was approved by the Institutional Review Board at OHSU (IRB #21775). Additional IRB approval was not required for this secondary analysis.

## Supporting information




**Supplemental Figure 1**. Flow chart of patient inclusion.

Supporting Information

## Data Availability

The data used in this analysis are stored securely in REDCap hosted at Oregon Health & Science University and the Dexcom Clarity Provider Portal. The data that support the findings of this study are not available upon request.
