# Supplementary material for: Continuous glucose monitoring profiles during postpartum glucose tolerance test results among people with gestational diabetes
Source: Pregnancy (Hoboken). 2025 Oct 27;1(6):e70143. doi: 10.1002/pmf2.70143 (PMC13344718; doi:10.1002/pmf2.70143)
Supplement: Supplementary file 1 [file PMF2-1-e70143-s002.docx]

**Supplemental Table 1.** Participant characteristics between those included vs. not included in sub-analysis

|  | All participants (n = 108) | Included (n = 51) | Excluded (n = 57) | *P* value |
| --- | --- | --- | --- | --- |
| Age | 32.9 ± 5.1 | 34.7 ± 3.9 | 31.3 ± 5.6 | <0.001 |
| Nulliparous (n (%)) | 44 (47.2) | 19 (37.3) | 25 (43.9) | 0.616 |
| BMI ≥ 30 (n (%)) | 65 (59.3) | 31 (60.8) | 34 (59.6) | >0.999 |
| Insurance (n (%)) |  |  |  | 0.256 |
| Private | 67 (62.0) | 35 (68.6) | 32 (56.1) |  |
| Public/other | 41 (38.0) | 16 (31.4) | 25 (43.9) |  |
| Early HbA1C | 5.3 ± 0.4 | 5.4 ± 0.5 | 5.3 ± 0.4 | 0.370 |
| Pregnancy Mean Glucose | 104.9 ± 12.0 | 103.3 ± 14.0 | 106.4 ± 9.6 | 0.210 |
| % Pregnancy TIR (63-140) | 91.3 ± 9.5 | 91.6 ± 11.6 | 90.9 ± 7.1 | 0.697 |
| % Pregnancy TAR (140) | 7.7 ± 9.7 | 7.1 ± 11.7 | 8.2 ± 7.3 | 0.566 |

Data presented in mean ± SD unless otherwise stated.
